# The molecular biology of memory: cAMP, PKA, CRE, CREB-1, CREB-2, and CPEB

**DOI:** 10.1186/1756-6606-5-14

**Published:** 2012-05-14

**Authors:** Eric R Kandel

**Affiliations:** 1 Department of Neuroscience, Columbia University, 1051 Riverside Drive, #664, New York, NY, 10032, USA

## Abstract

The analysis of the contributions to synaptic plasticity and memory of cAMP, PKA, CRE, CREB-1, CREB-2, and CPEB has recruited the efforts of many laboratories all over the world. These are six key steps in the molecular biological delineation of short-term memory and its conversion to long-term memory for both implicit (procedural) and explicit (declarative) memory. I here first trace the background for the clinical and behavioral studies of implicit memory that made a molecular biology of memory storage possible, and then detail the discovery and early history of these six molecular steps and their roles in explicit memory.

## Background: Simple systems in the study of implicit learning and memory

By 1969, we had already learned from the pioneering work of Brenda Milner that certain forms of memory were stored in the hippocampus and the medial temporal lobe. In addition, the work of Larry Squire revealed that there are two major memory systems in the brain: explicit or declarative; implicit or procedural. Explicit memory, a memory for facts and events—for people, places, and objects—requires, as Milner has pointed out, the medial temporal lobe and the hippocampus [1-3]. By contrast, we knew less about the localization of implicit memory, a memory for perceptual and motor skills and other forms of procedural memory which proved to involve not one but a number of different brain systems: the cerebellum, the striatum, the amygdala, and in the most elementary instances, simple reflex pathways themselves. Moreover, we knew even less about the mechanisms of any form of memory storage. Indeed, we did not even know whether the storage mechanisms were synaptic or non-synaptic.

In 1968, Alden Spencer and I were invited to write a perspective of learning for *Physiological Reviews*, which we entitled “Cellular Neurophysiological Approaches in the Study of Learning.” [4] In it we pointed out that there was no frame of reference for studying memory because one could not yet distinguish, experimentally, between the two conflicting approaches to the biology of memory that had been advanced up to that time: the *aggregate field approach* advocated by Karl Lashley in the 1950s and by Ross Adey in the 1960s, which assumed that information is stored in the *bioelectric field* generated by the aggregate activity of many neurons; and the *cellular connectionist approach*, which derived from Santiago Ramon y Cajal's idea [5] which postulated that learning results from changes in the strength of the synapse. The cellular-connection idea was later renamed synaptic plasticity by Konorski in his 1948 book: *Conditioned Reflexes and Neuronal Organization*[6]. Konorski’s idea was incorporated into a more specific model of certain types of learning by Hebb in 1949. Spencer and I concluded our review by emphasizing the need to develop tractable behavioral systems in which one could distinguish between these alternatives by relating, in a causal way, specific changes in the neuronal components of a behavior to modification of that behavior during learning and memory storage [4].

The first behavioral systems to be analyzed in this manner were simple forms of learning in the context of implicit memory. From 1964 to 1979, several useful model systems emerged: the flexion reflex of cats, the eye-blink response of rabbits, and a variety of simple forms of reflex learning in invertebrates: the gill-withdrawal reflex of *Aplysia*, olfactory learning in the fly, the escape reflex of *Tritonia*, and various behavioral modifications in *Hermissenda, Pleurobranchaea,* and *Limax*, crayfish, and honeybees. The studies were aimed at defining, then pinpointing, the neural circuits that mediate these behaviors and the critical synaptic sites within these circuits that are modified by learning and memory storage, and then specifying the cellular basis for those changes [7-14].

A number of insights rapidly emerged from this simple systems approach. The first was purely behavioral and revealed that even animals with a limited numbers of nerve cells—approximately 20,000 in the central nervous systems of *Aplysia* to 300,000 in *Drosophila*—have remarkable learning capabilities. In fact even the gill-withdrawal reflex, perhaps the simplest behavioral reflex of *Aplysia*, can be modified by five different forms of learning: habituation, dishabituation, sensitization, classical conditioning, and operant conditioning [15].

The availability of these simple systems opened up the first analyses of the mechanisms of memory, which focused initially on short-term changes lasting from a few minutes to an hour. These studies found that one mechanism for learning and short-term memory evident in both the gill-withdrawal reflex of *Aplysia* and in the tail flick response of crayfish is a change in synaptic strength brought about by modulating the release of transmitter. A decrease in transmitter release is associated with short-term habituation whereas an increase in transmitter release occurs during short-term dishabituation and sensitization ( [16-20]; for early reviews, see [21,22]).

Studies of memory in invertebrates also delineated a family of psychological concepts paralleling those first described in vertebrates by the classical behaviorists (Pavlov and Thorndike) and their modern counterparts (Kamin, Rescorla, and Wagner). These concepts include the distinction between various forms of associative and nonassociative learning and the insight that *contingency* – that the conditioned stimulus, in associative learning, is predictive of the unconditional stimulus - is more critical for learning than mere contiguity: the CS preceding the US (for review see [23]). Here, for the first time, psychological concepts, which had been inferred from purely behavioral studies, could be explained in terms of their underlying cellular and molecular mechanisms. For example, the finding that the same sensory to motor neuron synapses that mediate the gill-withdrawal reflex are the cellular substrates of learning and memory illustrates that the storage of procedural memory does not depend on specialized, superimposed memory neurons whose only function is to *store* rather than process information. Rather, the capability for simple procedural memory storage is built into the neural architecture of the reflex pathway.

## Emergence of a molecular biology of memory-related synaptic plasticity

### The delineation of cAMP and PKA in short-term memory storage

Cell biological studies of the synaptic connections between the sensory and motor neurons of the gill-withdrawal reflex in *Aplysia* revealed a biochemical mechanism for the short-term increase in transmitter release produced by sensitization [24] and later for classical conditioning (Hawkins et al., [25]). A single noxious (sensitizing) stimulus to the tail of *Aplysia* leads to the activation of three known classes of modulatory neurons, the most important of which uses the modulatory transmitter serotonin [26-28]. Serotonin stimulates the increase in synaptic strength produced by sensitizing stimuli to the tail. In 1976 Brunelli et al., [24] found that serotonin increases the level of cAMP in the sensory neurons.

cAMP (Cyclic Adenosine Monophosphate) had been discovered in 1958 by Earl Sutherland of Case Western Reserve as an intracellular “second” messenger that is activated in response to certain hormones – the “first” messengers – such as epinephrine, that by themselves cannot pass through the cell membrane [29]. For this discovery, Sutherland was awarded the Nobel Prize in Physiology or Medicine in 1971 [29]. To test the idea that serotonin produces its effects on sensitization by means of cAMP, Brunelli et al. [24] next injected cAMP directly into the sensory neurons and found that this resultant increase in the level of cAMP indeed led to a transient enhancement of transmitter release in the synaptic connection between the sensory and motor neuron of the gill-withdrawal reflex.

By the time Brunelli et al. did their first experiment, it was already known that cAMP mediates almost all of its actions through a kinase – an enzyme that phosphorylates proteins – called the cAMP-dependent protein kinase or protein kinase A (PKA). PKA had been discovered in 1968, a decade after the discovery of cAMP by Sutherland, by Edwin Krebs and Edmond Fischer, thereby establishing a mechanism not only for cAMP, but also a class of general mechanisms for second messenger signaling inside of cells [30]. In fact, PKA has served as a prototype for understanding other protein kinases and for appreciating the role of phosphorylation and dephosphorylation as a rapid and reversible means of modifying the activity of proteins. Krebs was also able to establish that the enzyme PKA is made up of four subunits: two regulatory subunits that inhibit two catalytic subunits. The catalytic subunits are the active phophorylating portions of the enzyme. When the level of cAMP rises in cells, the cAMP binds to the regulatory subunits of PKA, causing them to undergo a conformational change that frees the active catalytic subunits and allows it to phosphorylate its substrates. For this work, Fischer and Krebs were awarded the Nobel Prize in Physiology or Medicine in 1992. In 1980 Castellucci et al. [17] injected the catalytic subunit of PKA directly into the sensory neurons, and found that this was also sufficient to enhance transmitter release at the synaptic connection between the sensory neuron and the motor neuron.

### Classical conditioning involves both pre- and postsynaptic mechanisms of plasticity

In 1983, Hawkins, Abrams, Carew and Kandel [25] succeeded in establishing differential classical conditioning of the gill-withdrawal reflex and in beginning its cellular analysis. They found that paired training in which the CS (stimulation of the siphon) occurred just before the US (a shock to the tail) produced a greater increase in the withdrawal reflex than unpaired, CS alone, or US alone sensitization training. Compared with unpaired training, paired training also produced a greater increase in evoked firing of the motor neuron and greater facilitation of the monosynaptic connections between the sensory and the motor neurons. The reflex can also be differentially conditioned. When a conditioned stimulus is applied to two sites – the siphon and the mantle shelf – and used as a discriminative stimulus, only the paired CS is enhanced.

Further experiments indicated that this behavioral conditioning is due – in part – to activity-dependent amplification of presynaptic facilitation, which involves a differential increase in transmitter release from paired as compared to unpaired sensory neurons [15,25,31]. In addition there is a post-synaptic contribution [32-35]. Later experiments by Tom Abrams showed that Ca^2+^ influx during the paired spike activity enhances the activity of the adenyl cyclase, the enzyme in the sensory neuron that synthesizes cAMP [15,36]. This enzyme increases cAMP in response to serotonin. If serotonin arrives at a time when the Ca^2+^ level in the cell is increased by activity, the serotonin will lead to an enhanced synthesis of cAMP by the adenyl cyclase.

## A molecular biology of learning-related long-term synaptic plasticity

Beginning in 1980, the insights and methods of molecular biology were being brought to bear on the nervous system, making it possible to see commonalities in the molecular mechanisms of short-term memory among different animals and to begin to explore how short-term memory is converted to long-term memory.

Even earlier, in 1974, Seymour Benzer and his students had discovered that *Drosophila* can learn fear and that mutations in single genes interfere with short-term memory. Moreover, flies with these mutations do not respond to either classical conditioning of fear or to sensitization, suggesting that – as in *Aplysia* – the two types of learning have some genes in common [8,12]. Consistent with this idea Duncan Byers, Ron Davis, and Benzer found in 1981 that in most of the mutant flies, the genes identified represented one or another component of the cAMP pathway [37], the same pathway underlying sensitization and classical conditioning in *Aplysia*.

Studies of the gill-withdrawal reflex further revealed that even elementary forms of learning have distinct short- and long-term stages of memory storage. Whereas one training trial gives rise to a short-term memory lasting minutes, repeated spaced training gives rise to long-term memory lasting days to weeks [38,39]. These behavioral stages of synaptic plasticity were soon found to have parallels in the stages of the underlying synaptic plasticity – a short-term form lasting minutes to hours and a long-term form lasting days to weeks [40,41].

The simplicity of the neuronal circuit underlying the behavioral modifications of the gill-withdrawal reflex – including direct monosynaptic connections between identified mechanoreceptor sensory neurons and their follower cells [16,19] - has allowed reduction of the analysis of the short- and long-term memory for sensitization to the cellular and molecular level. This monosynaptic sensory to motor neuron connection, which is glutamatergic [42,43], can be reconstituted in dissociated cell culture [44]. This simplified *in vitro* model system reproduces what is observed during behavioral training by replacing the tail shocks with brief applications of serotonin, a modulatory transmitter normally released by sensitizing stimuli in the intact animal [26-28]. A single brief application of serotonin produces a short-term change in synaptic effectiveness (short-term facilitation or STF), whereas repeated and spaced applications produce changes in synaptic strength that can last for more than a week (long-term facilitation or LTF) [44]. The facilitation is also larger and longer lasting if as in classical conditioning the presynaptic sensory neuron fires action potentials just before the serotonin application, consistent with this mechanism for classical conditioning [45-47].

The first clue to how short-term memory is switched to long-term memory came when Louis Flexner, followed by Bernard Agranoff and his colleagues and by Samuel Barondes and Larry Squire, observed that the formation of long-term memory requires the synthesis of new protein.

As we have seen, activation of the of *Aplysia* sensory neurons by serotonin or by tail stimuli leads to a local increase in cAMP and the activation of the cAMP-dependent protein kinase A (PKA) by causing the catalytic subunits of this enzyme to dissociate from the regulatory subunits. The catalytic subunits can then phosphorylate different substrates in the synaptic terminals, such as potassium channels and proteins involved in exocytosis, leading to enhanced transmitter availability and release as described above for the storage of short-term memory. When serotonin stimulation is repeated a number of times, it causes a more persistent increase in the level of cAMP that leads to longer lasting forms of synaptic plasticity. This more robust pattern of stimulation causes the catalytic subunit of PKA to recruit p42 MAPK and both then move to the nucleus where they phosphorylate transcription factors and activate gene expression required for the induction of long-term memory [48,49].

In addition to protein kinases, synaptic protein phosphatases also play a key role in regulating the initiation of long-term synaptic changes. Various protein phosphatases, such as PP1 and calcineurin, counteract the local activity of PKA acting as inhibitory constraints of memory formation. For example, calcineurin can act as a memory suppressor for sensitization in the *Aplysia*[50]. Thus, an equilibrium between both kinase and phosphatase activities at a given synapse gates the synaptic signals that reach the nucleus and thus, can regulate both memory storage and retrieval [50].

### Activation of nuclear transcription factors

Long-term memory is represented at the cellular level by activity-dependent modulation of both the function and the structure of specific synaptic connections that, in turn, depends on the activation of specific patterns of gene expression [15]. As mentioned above, the inhibition of transcription or translation blocks the formation of long-term memory in a variety of model systems, but does not affect short-term memory.

Studies in *Aplysia* first revealed the participation of the cAMP/PKA-signaling pathway in synaptic facilitation and sensitization [15,24]. In 1986, Marc Montminy and R.H. Goodman first defined a conserved DNA sequence in the promoter elements that are activated by cAMP, then called CRE – the cAMP Response Element. The CRE is one of the DNA response elements contained within the control region of a gene. The binding of different transcription factors to these response elements regulates the activity of RNA polymerase, thereby determining when and to what level a gene is expressed. In 1987 Marc Montminy and L.M. Bilezikjian described CREB (cAMP Response Element Binding protein) as a cellular transcription factor that binds the cAMP response element (CRE) – thereby increasing the transcription of the somatostatin gene. The CRE-binding protein (CREB1), functions as a transcriptional activator only after it is phosphorylated by either PKA, MAPK or CaMK (for review see [51].) Evidence for a direct role of CRE-driven transcription, downstream of the cAMP pathway, in memory-related synaptic plasticity was provided more than a decade later. In 1990, Pramod Dash found that, during LTF in *Aplysia* neurons, PKA activates gene expression via an *Aplysia* CREB (Dash, Hochner, and Kandel, [52]). Blocking the binding of CREB1 to its DNA response element selectively eliminated the long-term process. Dash et al. [52] microinjected CRE oligonucleotides into sensory neurons co-cultured with motor neurons. This oligonucleotide inhibits the function of CREB1 by binding to the CREB1 protein within the cell, thereby preventing it from binding to CRE sites in the regulatory regions of cAMP-responsive genes and activating gene expression. While injection of the CRE oligonucleotide had no effect on STF, it selectively blocked LTF.

Most of the upstream signaling cascade leading to CREB activation appears to be conserved through evolution, and many aspects of the role of CREB in synaptic plasticity described in invertebrates have also been observed in the mammalian brain, although the role of CREB in explicit forms of memory appears to be more complex than in implicit forms of memory in invertebrates (see reviews by [53,54]).

In *Aplysia* sensory neurons, the activity of ApCREB1 leads to the expression of several immediate-response genes, such as ubiquitin hydrolase, that stabilize STF [55], and the transcription factor CCAAT-box-enhanced binding-protein (C/EBP), whose induction has been shown to be critical for LTF [56]. This induced transcription factor (in concert with other constitutively expressed molecules such as ApAF, [57]) activates a second wave of downstream genes that lead to the growth of new synaptic connections. These genes represent only two of a family of physiological relevant examples of gene products generated by CREB activity.

Initial studies of the molecular switch from short-term to long-term memory in *Aplysia* and *Drosophila* focused on regulators like CREB-1 that promote memory storage. However, subsequent studies in *Aplysia* and in the fly revealed the surprising finding that the switch to long-term synaptic change and the growth of new synaptic connections is also constrained by *memory suppressor genes* (see [58]). One important memory suppressor gene that constrains the growth of new synaptic connections is CREB-2 [58,59], which when over-expressed blocks long-term synaptic facilitation in *Aplysia*. When CREB-2 is removed, a *single* exposure to serotonin, which normally produces an increase in synaptic strength lasting only minutes, will increase synaptic strength for days and induce the robust growth of new synaptic connections [59].

The formation of LTF thus requires the activation of ApCREB1 by PKA [48] and the concomitant down-regulation of ApCREB2 by MAPK [49,60,61]. Conversely, the injection of pApCREB1 can by itself trigger facilitation lasting 24 h and this can be stabilized by a single pulse of serotonin [62,63].

These studies reveal that long-term synaptic changes are governed by both positive and negative regulators, and that the transition from STF to LTF requires the simultaneous removal of transcriptional repressors and activation of transcriptional activators. These transcriptional repressors and activators can interact with each other both physically and functionally. It is likely that the transition is a complex process involving temporally distinct phases of gene activation, repression, and regulation of signal transduction [58,62,63]. The CREB-mediated response to extracellular stimuli can be modulated by a number of kinases (PKA, CaMKII, CaMKIV, RSK2, MAPK, and PKC) and phosphatases (PP1 and Calcineurin). The CREB regulatory unit may therefore serve to integrate signals from various signal transduction pathways. This ability to integrate signaling, as well as to mediate activation or repression, may explain why CREB is so central to memory storage.

Finally, although we have focused on CREB- dependent gene expression is present in flies given its conserved role in memory formation through evolution, other transcription factors, such as SRF, c-fos, EGR-1 or NF-κB [64-67] are also likely to contribute to the transcriptional regulation that accompanies long-lasting forms of synaptic plasticity for different forms of learning in different animal species.

### Chromatin alteration and epigenetic changes in gene expression with memory storage

Studies by Guan et al. [68] have examined directly the role of CREB-mediated responses in long-term synaptic integration by studying the long-term interactions of two opposing modulatory transmitters important for behavioral sensitization in *Aplysia*. Using chromatin immunoprecipitation techniques to investigate how opposing inputs are integrated in the nucleus of sensory neurons, they found that both the facilitatory and inhibitory modulatory transmitters activate signal transduction pathways that alter promoter occupancy by activator or repressor CREB isoforms and subsequently affect nucleosome structure bidirectionally through acetylation and deacetylation of histone residues in chromatin.

These studies also revealed the contribution of histone tail acetylation, a modification that favors transcription and is associated with active *loci*, to LTF formation. Guan et al. [68] found that both facilitatory and inhibitory stimuli bidirectionally alter the acetylation stage and structure of promoters driven by the expression of genes involved in the maintenance of LTF, such as C/EBP. This study also demonstrated that enhancing histone acetylation with deacetylase (HDAC) inhibitors facilitates the induction of LTF [68]. These results indicate that critical chromatin changes occur during the formation of long-term memory and that these changes are required for the stable maintenance of these memories.

Although, epigenetic mechanisms were widely known to be involved in the formation and long-term storage of cellular information in response to transient environmental signals, the discovery of their putative relevance in adult brain function is relatively recent [68,69]. The epigenetic marking of chromatin, such as histone modification, chromatin remodeling and the activity of retrotransposons, may thus have long-term consequences in the transcriptional regulation of specific *loci* involved for long-term synaptic changes [70].

Long-term memory fundamentally differs from the short-term process in involving the growth of new synaptic connections. In one of the most surprising and dramatic findings in the early study of long-term memory, Craig Bailey and Mary Chen found that profound structural changes accompany the storage of long-term memory in both habituation and sensitization of the gill-withdrawal reflex. The sensory neurons from habituated animals retract some of their presynaptic terminals so that they make 35 percent fewer connections with motor neurons and interneurons than do sensory neurons from control animals. By contrast, following long-term sensitization the number of presynaptic terminals of the sensory neurons increases over two-fold. This learning-induced synaptic growth is not limited to sensory neurons. The dendrites of the postsynaptic motor neurons also grow and remodel to accommodate the additional sensory input. These results demonstrate that clear structural changes in both the pre-and postsynaptic cells can accompany even elementary forms of learning and memory in *Aplysia* and serve to increase or decrease the total number of functional synaptic connections critically involved in the behavioral modification [71]. Moreover, the findings on sensitization also provide evidence for an intriguing notion – that the growth of new synapses may represent the final and perhaps most stable phase of long-term memory storage, raising the possibility that the persistence of the long-term process might be achieved, at least in part, because of the relative stability of synaptic structure.

Together, these early cellular studies of simple behaviors provided direct evidence supporting Ramon y Cajal’s suggestion that synaptic connections between neurons are not immutable but can be modified by learning, and that those anatomical modifications serve as elementary components of memory storage. In the gill-withdrawal reflex, changes in synaptic strength occurred not only in the connections between sensory neurons and their motor cells but also in the connections between the sensory neurons and the interneurons. Thus, memory storage, even for elementary procedural memories is distributed among multiple sites. The studies showed further that a single synaptic connection is capable of being modified in opposite ways by different forms of learning, and for different periods of time ranging from minutes to weeks for different stages of memory.

## Synaptic capture

### Retrograde signaling from the synapse to the nucleus

One of the features that fundamentally distinguishes the storage of long-term memory from short-term cellular changes is the requirement for the activation of gene expression. Given this requirement at the nucleus, one might expect that LTF would have to be cell-wide. However, experiments by Martin et al. using local applications of serotonin in the *Aplysia* bifurcated sensory neuron-two motor neuron culture preparation [63,72], as well as parallel experiments by Frey and Morris in the hippocampus [73], demonstrated that synapses could be modified independently in a protein synthesis–dependent manner. Thus, LTF and the associated synaptic changes are synapse-specific, and this synapse specificity also requires CREB-1 and is blocked by an antibody to CREB-1. This implies that there must be not only retrograde signaling from the synapse back to the nucleus [72,74], but also anterograde signaling from the nucleus to the synapse. Recently, Thompson et al. [75] have found that serotonin stimulation which produces LTF in *Aplysia* sensory-motor neuron co-cultures triggers the nuclear translocation of importins, proteins involved in carrying cargos through nuclear pore complexes (see also [74]). Similarly, in hippocampal neurons, NMDA activation or LTP induction, but not depolarization, leads to translocation of importin [75]. Although details underlying the translocation of these retrograde signals remain unknown, the effector molecules identified thus far appear to be conserved in both invertebrates and vertebrates. The future identification of the molecular cargoes of importin and its signaling role in the nucleus are likely to increase our understanding of how transcription-dependent memory is regulated.

Following transcriptional activation, newly synthesized gene products, both mRNAs and proteins, have to be delivered specifically to the synapses whose activation originally triggered the wave of gene expression. To explain how this specificity can be achieved in a biologically economical way in spite of the massive number of synapses in a single neuron, Martin et al. [49,61,72] and Frey and Morris [73] proposed the synaptic capture hypothesis. This hypothesis, also referred to some times as synaptic tagging, proposes that the products of gene expression are delivered throughout the cell, but are only functionally incorporated in those synapses that have been tagged by previous synaptic activity. The “synaptic tag” model has been supported by a number of studies both in the rodent hippocampus [73,76-78] and *Aplysia *[63,72].

### Molecular mechanisms of synaptic capture

Studies of synaptic capture at the synapses between the sensory and motor neurons of the gill-withdrawal reflex in *Aplysia* have demonstrated that to achieve synapse-specific LTF more than the production of CRE-driven gene products in the nucleus is necessary. One also needs a PKA-mediated covalent signal to mark the stimulated synapses and local protein synthesis to stabilize that mark [63,72]. Thus, injection into the cell body of phosphorylated CREB-1 gives rise to LTF at all the synapses of the sensory neuron by seeding these synapses with the protein products of CRE-driven genes. However, this facilitation is not maintained beyond 24–48 hours and not accompanied by synaptic growth unless the synapse is also marked by the short-term process, a single pulse of serotonin [63].

How is a synapse marked? Martin et al. [72] found two distinct components of marking in *Aplysia*, one that requires PKA and initiates long-term synaptic plasticity and growth, and one that stabilizes long-term functional and structural changes at the synapse and requires (in addition to protein synthesis in the cell body) local protein synthesis at the synapse. Since mRNAs are made in the cell body, the need for the local translation of some mRNAs suggests that these mRNAs are presumably dormant while they are transported from the cell body to the synapses of the neuron and are only activated at appropriate synapses in response to specific signals. If that were true, one way of activating protein synthesis at these specific synapses would be to recruit to these synapses a regulator of translation that is capable of activating dormant mRNA.

Kausik Si began to search for such a regulator of protein synthesis. In *Xenopus* oocytes, Joel Richter had found that maternal RNA is silent until activated by the cytoplasmic polyadenylation element binding protein (CPEB) [79]. Si searched for a homolog in *Aplysia* and found in addition to the developmental isoform studied by Richter a new isoform of CPEB with novel properties. Blocking this isoform at a marked (active) synapse prevented the maintenance but not the initiation of long-term synaptic facilitation [80,81]. Indeed, blocking ApCPEB blocks memory days after it is formed. An interesting feature about this isoform of *Aplysia* CPEB is that its N-terminus resembles the prion domain of yeast prion proteins and endows similar self-sustaining properties to *Aplysia* CPEB. But unlike other prions which are pathogenic, ApCPEB appears to be a functional prion. The active self-perpetuating form of the protein does not kill cells but rather has an important physiological function.

The Si lab and the Barry Dickson lab have found, independently, that long-term memory in *Drosophila* also involves CPEB for a learned courtship behavior in which males are conditioned to suppress their courtship upon prior exposure to unreceptive females. When the prion domain of the *Drosophila* CPEB is mutated, there is loss of long-term courtship memory [82,83].

Prion-like proteins represent auto-replicative structures that may serve as a persistent form of information [84]. Si and I have recently proposed a model based on the prion-like properties of *Aplysia* neuronal cytoplasmic polyadenylation element binding protein (CPEB) [85]. Neuronal CPEB can activate the translation of dormant mRNAs through the elongation of their poly-A tail. *Aplysia* CPEB has two conformational states: one is inactive or acts as a repressor, while the other is active. In a naive synapse, the basal level of CPEB expression is low and its state is inactive or repressive. According to the model of Si et al., serotonin induces an increase in the amount of neuronal CPEB. If a given threshold is reached, this causes the conversion of CPEB to the prion-like state, which is more active and lacks the inhibitory function of the basal state [85]. Once the prion state is established at an activated synapse, dormant mRNAs, made in the cell body and distributed cell-wide, would be translated but only at the activated synapses. Because the activated CPEB can be self-perpetuating, it could contribute to a self-sustaining synapse-specific long-term molecular change and provide a mechanism for the stabilization of learning-related synaptic growth and the persistence of memory storage.

## Emergence of a genetics of learning-related synaptic plasticity for explicit memory

Unlike implicit memory, the conscious remembrance of things past requires a complex system involving the medial temporal lobe and the hippocampus. A new era of research was opened in 1971 when John O’Keefe made the amazing discovery that neurons in the hippocampus of the rat register information not about a single sensory modality—sight, sound, touch, or pain—but about the space surrounding the animal, a feat that depends on information from several senses (O'Keefe and Dostrovsky, [85]). These cells which O'Keefe referred to as "place cells" fire selectively when an animal enters a particular area of the spatial environment. Based on these findings, O’Keefe and Nadel [86] suggested that the hippocampus contains a cognitive map of the external environment that the animal uses to navigate.

Independent of O’Keefe, Timothy Bliss and Terje Lømo, working in Per Andersen’s laboratory in Oslo, were also investigating the hippocampus and discovered that the synapses of the perforant pathway of the hippocampus have remarkable plastic capabilities that could serve for memory storage (Bliss and Lømo, [87]). It soon became clear that a brief, high frequency train of action potentials in any one of the three major hippocampal pathways strengthens synaptic transmission. This long-term potentiation (LTP) has several forms. In the perforant and Schaffer collateral pathways, LTP is associative, requiring presynaptic closely followed by postsynaptic activity. In the mossy fiber pathway, LTP is nonassociative; it requires no coincident activity [88].

A key insight into the various forms of LTP derived from Jeffrey Watkins’s discovery in the 1960s that glutamate is the major excitatory transmitter in the brain and that it acts on a number of different receptors, which he divided into two major groups: NMDA and non-NMDA (AMPA, kainate, and metabotropic) receptors. In the course of finding specific antagonists for each of these, Watkins discovered that Mg^2+^ blocks the NMDA receptor [89]. Philippe Ascher and independently Gary Westbrook next found that the Mg^2+^ blockade is voltage-dependent [90,91]. This was important because LTP in the Schaffer collateral pathway requires the NMDA receptor [89] and the receptor is unblocked when the postsynaptic cell is depolarized, which normally occurs only in response to a burst of presynaptic action potentials. Thus, the NMDA receptor has Hebbian associative properties; to release the Mg^2+^ blockade, the presynaptic neuron must be activated to provide glutamate just before the postsynaptic cell fires an action potential [89].

Gary Lynch and Roger Nicoll next found that the induction of LTP in the Schaffer collateral pathway requires an influx of Ca^2+^ into the postsynaptic cell [92,93]. The Ca^2+^ activates directly or indirectly at least three protein kinases: (1) calcium/calmodulin protein kinase II [94,95]; (2) protein kinase C [96]; and (3) the tyrosine kinase fyn [97,98].

A major question that dominated thinking in the 1980s and 1990s is whether LTP in the Schaffer collateral pathway is expressed pre- or postsynaptically. Nicoll’s finding that LTP in the Schaffer collateral pathway is associated with a selective increase in the AMPA-type receptor component of the EPSP with little change in the NMDA-type receptor component provided the first evidence that LTP at this synapse is both initiated and expressed postsynaptically [99]. Roberto Malinow and Nicoll indepdently discovered that the increase in response of the AMPA-type receptors is due to a rapid insertion of new clusters of receptors in the postsynaptic membrane from a pool of intracellular AMPA-type receptors stored in recycling endosomes [100-102]. Other studies, however, have implicated additional presynaptic changes that require one or more retrograde messengers from the postsynaptic cell [103,104]. These differences may depend on the frequency or pattern of stimulation used, or as suggested by Alan Fine, or on the developmental stage of the hippocampus [105].

In 1986 Richard Morris made the first connection of LTP to spatial memory [106] by demonstrating that NMDA receptors must be activated for spatial learning in the rat. This led to a more detailed set of correlations between memory and the phases of LTP.

a) *Earlier and Late LTP*

Long-term potentiation in the hippocampus proved to have both early and late phases, much as long-term synaptic facilitation in *Aplysia* does. One train of stimuli produces the early phase (E-LTP), which lasts 1 to 3 hours and does not require protein synthesis. Four or more trains induce the late phase (L-LTP), which lasts at least 24 hours, requires protein synthesis, and is activated by PKA [107,108].

Unlike the early phase, which can involve separate presynaptic or postsynaptic changes, the late phase depends on a coordinate structural change in both the presynaptic and postsynaptic cell through the action of one or more orthograde and retrograde messengers that assure the orderly and coordinated remodeling of both components of the synapse.

b) *Molecular Similarities Between Procedural and Declarative Memory*

Procedural and declarative memory differ dramatically at the behavioral and cellular level. They use a different logic (unconscious versus conscious recall) and they are stored in different areas of the brain. Nevertheless, as summarized above, the two types share in common several molecular steps and an overall molecular logic. Both are created in at least two stages: one that does not require the synthesis of new protein and one that does. In both, short-term memory involves covalent modification of preexisting proteins and changes in the strength of preexisting synaptic connections, while long-term memory requires the synthesis of new protein and the growth of new connections. Moreover, at least some examples of both forms of memory use PKA, MAP kinase, CREB-1, and CREB-2 signaling pathway for converting short-term to long-term memory. Finally, both forms appear to use morphological changes at synapses to stabilize long-term memory, and both forms require a synaptic tag [109].

In the 1980s and 1990s, genetic analyses of behavior pioneered in *Drosophila* by Seymour Benzer were opened up for the mouse by Ralph Brinster, Richard Palmiter, Mario Capecci, and John Smythies (for reviews see [110,111]). It soon became possible to selectively manipulate individual genes in an intact animal to compare the effects of such manipulations on long term memory on the one hand and on the other hand on different forms of LTP in isolated hippocampal slices. These techniques, first used to study memory by Alcino Silva in Susumu Tonegawa’s lab [112,113] and by Seth Grant in my lab [97], revealed that interfering with LTP by knocking out specific kinases (CAMKII, fyn) also interfered with spatial memory.

Genetically modified mice were also used to determine the consequences of selective defects in the late phase of LTP. Ted Abel developed transgenic mice that expressed a mutant gene that blocks the catalytic subunit of PKA [107]. Silva and Rusiko Bourtchouladze studied mice with mutations in CREB-1 [114]. Both lines of mice–those that blocked PKA with a knockout of CREB-1–had a serious defect in long-term spatial memory and both had roughly similar defects in LTP: the early phase was normal, but the late phase was blocked, providing strong evidence linking the phases of LTP to the phases of memory storage [107,114].

In addition, a homolog of *Aplysia* CPEB named CPEB-3 has been found in mice raising the possibility that CPEB-3 may perform a similar function in vertebrates [115]. A parallel, self-sustaining mechanism, mediated by PKMzeta, has been discovered independently in the mammalian brain by Todd Sacktor. Blocking PKC-zeta interferes with memory even days or weeks after it is formed [116]. The finding of the roles of CPEB-3 and PKCzeta in the maintenance of memory storage suggests that in mammals as well as in *Aplysia* and flies memory must be actively sustained for long periods of time.

c) *Consolidation and Competition in Memory*

Competition between neurons is necessary for refining neural circuitry, but does it play a role in encoding memories in the adult brain? In studies of the amygdala, Sheena Josselyn and Silva found that neurons with large amounts of the CREB switch, required for long-term memory, are selectively recruited in the memory of fear. Indeed, the relative activity of CREB at the time of learning determines whether a neuron is recruited [117]. Conversely, if such neurons are deleted after learning, the memory of fear is blocked [118].

Long-term memory requires also transcriptional activity in genes such as c-fos, zif268, that arc are rapidly and transiently induced by high frequency neural activity. Activity in these genes has therefore been used for many years to map brain activity patterns in the rodent brain. By providing a genetic readout of patterns of neural activity these genes provide the potential to obtain direct molecular control over ensembles of neurons based on their response to a given experience. In one recent study, the c-fos promoter was combined with elements of the Tet regulatory system in transgenic mice to allow the introduction of a lacZ marker into neurons activated with fear conditioning [119]. The marker provided a long-lasting record of brain activity during learning that could be compared to activity during recall. A partial reactivation of the neurons that were active during learning and the strength of the recalled memory were correlated with the degree of circuit reactivation. This important new approach provides an opportunity to introduce any genetically encoded effector molecule into neurons based on their recent activity thereby providing the potential to study circuits based on the specific memory they encode.

## Overall view

We now understand – in considerable molecular detail – the mechanisms underlying long-term learning-related synaptic plasticity, and the importance that such plastic changes play in memory storage, across a broad range of species and forms of memory. One surprising finding is the remarkable degree of conservation of the molecular memory mechanisms: cAMP, PKA, CRE, CREB-1 and CREB-2, and even CPEB, in different brain regions within a species and across species widely separated by evolution. In fact, one of the most striking features that has emerged through the application of molecular biology to neural science is the ability to see how unified all of the biological sciences have become.

However, although it is now clear that long-term synaptic plasticity is a key step in memory storage, it is important to note that a simple enhancement in the efficacy of a synapse is not sufficient to store a complex memory. Rather, changes in synaptic function must occur within the context of an ensemble of neurons to produce a specific alteration in information flow through a neural circuit. With the recent development of powerful genetic tools, it may soon be possible to meet the daunting challenge of visualizing and manipulating such changes in neural circuitry [120].

It also will be interesting to see to what degree computational models will contribute to our further understanding of synaptic plasticity. The influential cascade model of synaptically stored memory by Stefano Fusi, Patrick Drew, and Larry Abbott [121] emphasizes that switch-like mechanisms are good for acquiring and storing memory but bad for retaining it. Retention, they argue, requires a cascade of states, each more stable than its precursor. As their hypothesis predicted, a progressive stabilization of changes in the synapse has been found to take place during the transition from short-term to intermediate-term to long-term memory storage (Jin et al. [122,123]). Moreover, possible interactions between CPEB and PKC-ζ might provide additional semi-stable states within the long-term memory domain.

A major reason why computational neuroscience is rising and becoming more powerful and more interesting, as evident in the cascade model, is that these models lend themselves to experimental testing. In the future, however, computational models will need to broaden their focus to include the role of modulatory transmitters, the molecular components of synapses and their anatomical substrates.

Finally, we need to understand how memory is recalled. This is a deep problem whose analysis is just beginning. Mayford has made an important start of this problem and found that the same cells activated in the amygdala during the acquisition of learned fear are reactivated during retrieval of those memories. In fact, the number of reactivated neurons correlated positively with the behavioral expression of learned fear, indicating that associative memory has a stable neural correlate [119]. But one of the characteristics of declarative memory is the requirement for conscious attention for recall. How does this attention mechanism come into play? Do modulatory transmitters such as dopamine and acetylcholine have a role in the retrieval process?
